# End-to-End 3-D Sound Source Localization from the Raw Waveform Based on Stereo Microphone Array

**DOI:** 10.3390/s26082372

**Published:** 2026-04-12

**Authors:** Lipeng Xu, Chao Yang

**Affiliations:** School of Mechanical and Automotive Engineering, Shanghai University of Engineering Science, Shanghai 201600, China; jxqcxy@sues.edu.cn

**Keywords:** sound source localization, end-to-end, convolutional neural networks, tetrahedral stereo microphone array, residual connection, attention mechanism

## Abstract

The problem of performance degradation in current sound source localization algorithms under reverberant and noisy environments remains a critical challenge. Consequently, this paper introduces a novel approach to estimate the 3-D position of sound sources directly from raw audio signals using an artificial neural network (ANN), which improves the performance of sound source localization algorithms under reverberant and noisy environments. Instead of relying on handcrafted features, raw audio signals recorded by a tetrahedral stereo microphone array are fed directly into the ANN. This design eliminates spatial symmetry issues found in 2-D microphone arrays and enhances 3-D localization accuracy. Inspired by human auditory systems, a convolutional layer is added after the input layer to simulate frequency analysis to search localization cues in different frequency bands. Furthermore, the proposed algorithm incorporates residual connections (RC) and squeeze-and-excitation (SE: an attention mechanisms). Residual connections introduce raw features into deeper network layers to prevent localized information loss caused by excessive network depth, while also enabling improved model training stability. The attention mechanism dynamically adjusts weights across and within channels, suppressing interference while enhancing localization-critical cues, thereby playing a pivotal role in boosting the algorithm’s reverberation and noise resistance. Experimental results demonstrate significant improvements: in semi-anechoic chambers, the method reduces localization errors by 0.2 m and increases accuracy by 10%; in conference rooms, errors decrease by 0.26 m with a 21% accuracy gain. These outcomes conclusively validate the effectiveness of the proposed approach in enhancing robustness against reverberation and noise in sound source localization systems.

## 1. Introduction

In the last decade, much effort has been spent on the development of sound source localization (SSL) technology. SSL pertains to the problem of determining the spatial location of a sound source using only the signals captured by a microphone array. The traditional approach involves signal processing-based algorithms, including the estimation of time differences of arrival (TDOA) between microphone pairs using techniques like generalized cross-correlation with phase transform (GCC-PHAT) [1,2,3], beamforming methods such as SRP-PHAT [4,5], and spectral estimation approaches like the multiple signal classification (MUSIC) algorithm [6,7]. However, with the development of research, the same issue arises in all of these classical algorithms: their performance will decrease dramatically when reverberation and noise are present simultaneously [6].

Therefore, researchers have turned to machine learning algorithms as a potential solution [8]. In recent years, numerous sound source localization algorithms based on deep learning have been developed. In studies [1,9,10,11,12,13,14], inspired by traditional methods, researchers have used the time differences of arrival (TDOA) as input features to neural networks, leveraging the network’s nonlinear fitting capability to map these features to the sound source location. Their findings demonstrate that neural networks can effectively learn this mapping, improving the accuracy of azimuth angle estimation. However, these approaches are limited to estimating the source distance. In [15,16], both sound intensity and arrival time difference are combined as input features to the neural network. This approach shows that sound intensity is strongly correlated with the distance of the source, allowing for simultaneous estimation of both azimuth angle and distance. Despite these advances, these methods rely on two-dimensional microphone arrays, which suffer from the mirror effect and do not enable full three-dimensional localization.

Up until now, the methodologies discussed have relied on manually extracted features as inputs for neural networks, which present an inherent challenge. This approach limits us to using only those features that have been rigorously studied in the field of sound source localization, inadvertently overlooking potentially valuable characteristics that remain unexplored. Consequently, important information may be missed during the feature extraction phase, hindering the full utilization of available data and impeding progress in positioning accuracy. Drawing inspiration from the human auditory system’s natural ability to localize sound sources, an alternative strategy has emerged [17,18]. In this approach, neural network models are used to mimic the functional mechanisms of the human ear, eliminating the need for manual feature extraction. These models can directly determine the spatial orientation of sound sources from raw audio signals, to create an end-to-end sound source localization framework. This methodology not only aligns with the biological capabilities of human hearing but also represents a leading trend in the evolution of artificial neural networks, pushing toward more holistic and autonomous learning systems.

End-to-end systems have achieved substantial progress across diverse audio applications [17,19,20,21,22]. For example, the end-to-end sound source localization (SSL) model in [17] directly infers sound source azimuth from raw binaural waveforms, validating that end-to-end architectures markedly improve reverberation suppression and noise reduction; its integrated frequency analysis module further delivers state-of-the-art azimuth estimation accuracy, setting a new task benchmark.

Parallel progress has been made in audio synthesis, with end-to-end models that directly map text to raw waveforms [23,24]. These works validate end-to-end learning’s ability to align discrete text and continuous audio signals, underscoring this paradigm’s transformative potential for audio processing.

Although existing deep learning-based sound source localization methods have made substantial progress, they still face the following key challenges in practical applications:

1. Insufficient robustness against interference in complex acoustic environments: background noise, reverberation, and non-stationary interference can severely disrupt the spatial characteristics of the sound field, making it difficult for conventional neural networks to stably extract reliable azimuth information.

2. Inadequate fusion of spatial and spectral features: many methods process spectral and spatial features separately, neglecting the high-order interactions between them, which limits localization accuracy under low signal-to-noise ratio (SNR) conditions.

3. Weak adaptability of network structures to critical frequency bands: different frequency bands contribute differently to source localization, and existing methods lack effective channel attention mechanisms to dynamically enhance informative frequency bands while suppressing interfering ones.

This paper introduces a novel deep convolutional neural network (CNN) designed to directly infer the 3-D spatial coordinates of sound sources in a Cartesian framework from raw waveforms, eliminating manual feature extraction. Leveraging a stereo microphone array, the proposed model resolves spatial ambiguity. The architecture contains three core components: a frequency analysis module, a residual connection module, and a squeeze-and-excitation (SE) block. Inspired by [17], the frequency analysis module is integrated to enhance performance. Unlike conventional methods that simply stack convolutional layers, we introduce residual connections to capture both global waveform characteristics and fine-grained local features, thus improving localization accuracy. Furthermore, the SE block acts as a channel attention mechanism for dynamic feature weighting, which optimizes feature utilization and further boosts overall performance.

## 2. Materials and Methods

### 2.1. Data-Set

To ensure the exceptional performance of the deep learning network model, a substantial amount of customized training data is essential. The project requires multi-channel audio data captured within controlled acoustic environments, along with corresponding spatial labels for each recording. However, obtaining such specialized datasets from existing open-source repositories is impractical. As a result, a simulated approach has been adopted to generate the required audio samples.

The room impulse response (RIR), denoted by *h*(*t*), represents the impulse response of a linear time-invariant (LTI) system, which fully characterizes the propagation of sound waves from a source to a microphone (receiver). As a critical tool in acoustics, the RIR describes how sound waves propagate within enclosed spaces (e.g., rooms, concert halls). In free-field conditions, the acoustic pressure *p*(*r*, *t*) generated by a point source satisfies the wave equation:

(1) $$\nabla^{2}p-\frac{1}{c^{2}}\frac{\partial^{2}p}{\partial t^{2}}=-\delta (r-r_{0})\delta (t)$$

where *c* is the speed of sound, *δ* denotes the impulse (Dirac delta) function. The RIR of an enclosed space can be approximated using the Image Source Method (ISM):

(2) $$h(t)=\sum_{i=0}^{N}\frac{A_{i}}{\tau_{i}}\delta (T-\frac{r_{i}}{c})$$

where $r_{i}$ is the propagation distance of the *i*-th image source to the receiver, *T* denotes the propagation time of the acoustic wave, and $\tau_{i}$ denotes the time variable, $A_{i}$ is the reflection coefficient of the *i*-th path, and *N* denotes the number of significant reflections considered. For a microphone array with *M* elements, each microphone m has an independent room impulse response (RIR) h(t), and the captured signal $x_{m}(t)$ at the *m*-th microphone can be expressed as follows:

(3) $$x_{m}(t)=s(t)\times h_{m}(t)+n_{m}(t),m=1,2,3,\ldots ,M$$

where $x_{m}(t)$ is the signal captured by the *m*-th microphone, s(t) denotes the source signal, $h_{m}(t)$ is the RIR of the *m*-th microphone, and $n_{m}(t)$ corresponds to the noise at the *m*-th microphone.

**Figure 1:**
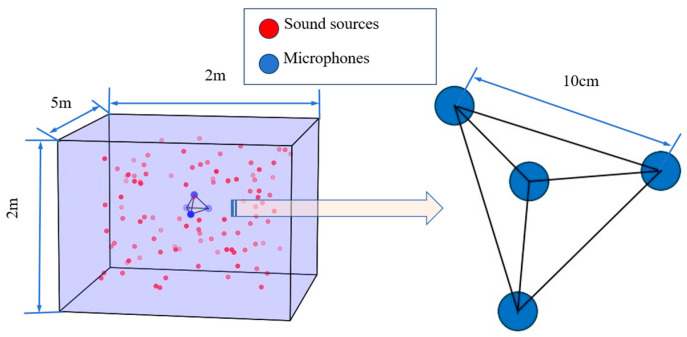
Room-set (**left**) and microphone array structure (**right**).

Using the Python 3.9 library pyroomacoustics, any desired acoustic environment can be carefully constructed, enabling the generation of the relevant audio datasets. The process begins with the creation of a virtual room tailored to the project’s specifications. By setting the desired reverberation time, the algorithm utilizes the ISM to model the reflection of the initial sound source across every reflective surface, to map the sound wave’s reflection paths. This detailed process effectively simulates the room’s early reflections and its unique acoustic characteristics. Next, both the sound source and microphone array (shown in Figure 1) are strategically positioned within the virtual room to simulate the transmission and reception of acoustic signals (also shown in Figure 1).

The algorithm then generates the RIR, capturing the propagation characteristics of sound within the designed environment. By convolving the RIR with selected audio signals, the model accurately simulates how the signals would propagate in the simulated room. The microphone array then captures these signals as multi-channel audio data. Each signal originates from a designated sound source location, propagates through the carefully designed acoustic space, and is captured by the microphone array. This process generates the specialized audio data needed for the proposed model. For the audio content, an open dataset named mini speech commands (https://storage.googleapis.com/download.tensorflow.org/data/mini_speech_commands.zip (accessed on 15 December 2025)) published by google, which containing 8800 clean, single-channel voice recordings from 100 different speakers, is used. These recordings consist of simple English phrases, such as “down” and “right”. From this extensive dataset, 80 unique voice samples are selected to form the basis of the training set. A summary of the dataset details is provided in Table 1.

To enhance the model’s robustness across various acoustic environments, three parameter values for signal-to-noise ratio, reverberation time and room size are chosen, resulting in 108 distinct acoustic environments that encompass nearly all real-world scenarios. Meanwhile, pink noise, as the most representative of real-world background noise, is mixed into speech signals at a defined signal-to-noise ratio (SNR). In each simulated environment, a four-microphone array is arranged in a tetrahedral shape, with microphones spaced 10 cm apart and the array’s center aligned with the room’s center. Sound sources are randomly distributed throughout the room, with a total of 10,000 sound source locations sampled at a 16,000 Hz. This setup generates 1080,000 four-channel voice data samples. To eliminate the influence of extraneous variables, the parameters of these variables were fixed as shown in Table 2. Reverberation causes long pure reverb fragments in the generated voice data, which are removed using voice silence detection techniques. This process reduces the size of the final dataset and facilitates faster model convergence during training.

The label data consists of the Cartesian coordinates (*X*, *Y*, *Z*) of the sound source within the room. The dataset is then stored as tensors in HDF5 file format. The shape of the voice data tensor is 1080,000 × 8000 × 4, where 1080,000 represents the number of data samples, 8000 represents the number of sampling points per audio sample, and 4 represents the number of channels. The shape of the label data tensor is 1080,000 × 3, where 1080,000 corresponds to the number of data samples and 3 corresponds to the three components of the Cartesian coordinates. The total dataset is divided into the training set and validation set at a ratio of 4:1.

### 2.2. Baseline Method

This approach capitalizes on the strengths of end-to-end frameworks, which have gained widespread recognition for their effectiveness across various fields [17]. By adopting this methodology, the study seeks to demonstrate its potential advantages in sound source localization. Several studies have highlighted the benefits of end-to-end systems, especially in simplifying model design and enhancing overall performance. Although this may be one of the attempts to apply end-to-end techniques to sound source localization, it builds on a growing body of research that supports the effectiveness of such approaches. This innovative application has the potential to significantly outperform traditional methods, particularly in terms of accuracy and computational efficiency.

**Figure 2:**
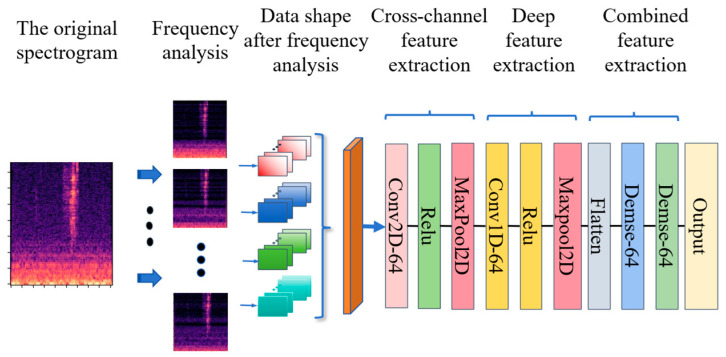
Baseline model structure.

This paper recalibrated the model with the aim of achieving three-dimensional localization determination by adjusting its input schema. As shown in Figure 2, the process begins with a one-dimensional convolutional layer for preliminary frequency decomposition, following a strategy similar to the advanced model’s initiation. The methodology proceeds with cross-channel feature extraction via a subsequent two-dimensional convolutional layer, followed by pooling for subsampling. To explore the feature hierarchy further, a secondary one-dimensional convolutional layer is employed, followed by ReLU activation to introduce non-linearity. Pooling is applied again for additional dimensionality reduction. The compacted information is then flattened and passed through two fully connected layers, a crucial stage for consolidating and refining these distilled features while reducing feature dimensionality. This rigorous pipeline culminates in the output layer providing the three-dimensional spatial coordinates. All hyperparameter settings strictly adhere to those outlined in the referenced literature, except for a single adjustment to the number of neurons in the fully connected layers (reduced from 1024 to 64, with the error less than 5%). This adjustment—carefully calculated to have minimal impact on model efficacy—serves to reduce computational complexity and align the model with the constraints of contemporary hardware architectures. Specifically, this recalibration is implemented by optimizing the channel number and tensor shape of each convolutional layer, with the core goal of balancing localization accuracy, model parameter size, and inference efficiency. The tensor shape of each layer after recalibration is detailed in Table 3.

### 2.3. Proposed Method

**Figure 3:**
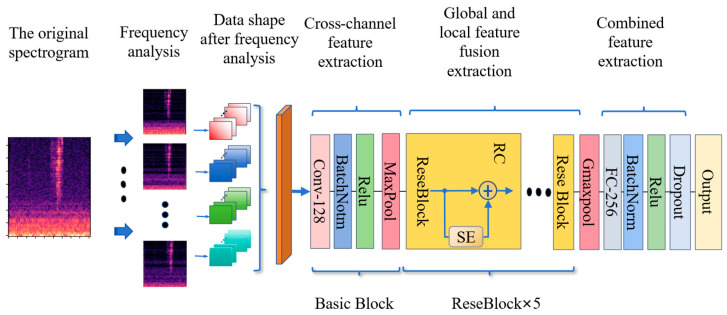
Proposed model structure.

The structure of the proposed end-to-end method is illustrated in Figure 3 and each layer’s tensor shape is shown in Table 4. The model is composed of several key modules: a basic block, a frequency analysis module, a residual module (RC, the yellow box in Figure 3), and an attention mechanism module (SE). Upon inputting data into the model, the frequency analysis module processes each of the four channels, simulating the human auditory system to produce frequency analysis results. Next, the residual module is applied for further feature extraction. The features across channels contain critical information about the sound source’s position. To extract positional cues between channels, the residual module employs two-dimensional convolution. Simultaneously, the attention mechanism module is used to dynamically adjust the importance of features across channels, improving the efficiency of feature representation in the model. Finally, the model uses a batch normalization layer and two fully connected layers to compute the final position coordinates of the sound source.

**Figure 4:**
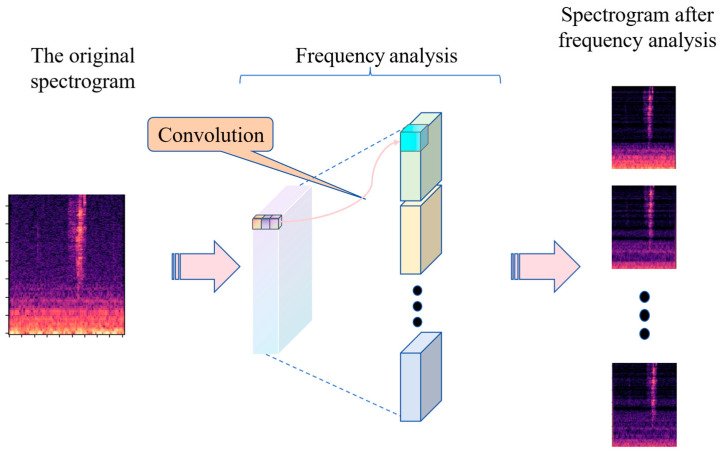
Frequency analysis of speech.

According to a key reference in auditory psychology [25], the human auditory system localizes sound sources by processing them through frequency-specific analysis. Specifically, the ear decomposes incoming sound signals into different frequency bands and analyzes each band independently. This frequency-specific processing allows for precise localization of sound sources by exploiting distinctive cues across various frequency ranges. Inspired by this mechanism, a frequency analysis module is introduced in this study to emulate the human ear’s process. The structure of the frequency analysis module is shown in Figure 4. It uses a convolutional layer with 64 1-D kernels, each with a shape of 1 × 256, serving as time-domain filters for frequency analysis. The convolutional kernels, directly trained on raw waveforms, are expected to closely resemble the sinusoidal components comprising the waveform. These convolutional kernels act as a set of sinusoidal functions, defining a unique frequency response for each kernel [17]. After batch normalization (a technique that accelerates model convergence) and ReLU activation (which enhances the model’s capacity to capture non-linearities), the output is passed forward. Notably, the standard convolutional module within this architecture incorporates 128 filters, a design decision that significantly increases the feature space dimensionality and, consequently, the model’s expressive power.

**Figure 5:**
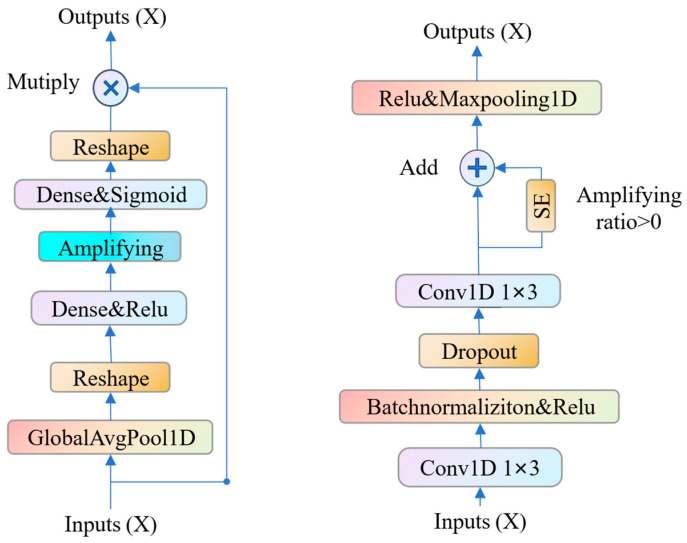
Squeeze-and-excitation block (**left**) and residual module (**right**).

As shown in Figure 5, the residual module begins with a primary feature extraction phase performed by a basic block. A parallel pathway is then introduced, where a portion of the data is routed through an advanced attention mechanism. This module dynamically adjusts the importance of different data channels, enhancing the model’s ability to capture subtle and intricate feature representations. The output from the attention mechanism is subsequently merged back with the main data flow through an additive combination, followed by ReLU activation to introduce non-linearity. Finally, pooling operations are applied to compress these features, distilling the most essential information and reducing computational redundancy.

## 3. Experiment and Analysis

### 3.1. Experimental Environment and Parameter Setting

The experimental setup utilized a Lenovo workstation () running the Windows 7 operating system, equipped with an 8-core Intel CPU (3.7 GHz), 32 GB RAM, and a 1-terabyte hard drive to ensure sufficient resources for data-intensive tasks.

Python was chosen as the programming language, with Keras (TensorFlow’s high-level API) employed to construct the neural network models. Keras simplified the management of hyperparameters, including the selection of the Adam optimizer, which is known for its efficiency in gradient-based learning algorithms. The learning rate was set to the default value of 0.001, balancing fast convergence and stability.

The Mean Squared Error (MSE) was selected as the loss function, as it is a commonly used metric for regression tasks, effectively capturing errors and ensuring dimensional consistency in error measurement. Root Mean Squared Error (RMSE) was used as the evaluation metric, providing an easily interpretable measure of the model’s predictive error. Given the hardware constraints, the batch size was set to 16 to optimize memory usage, and the model was trained for 200 epochs, balancing thorough training with computational feasibility. In order to further enhance the training process, callback functions were incorporated into the regimen to monitor progress dynamically. These functions enabled real-time tracking of training, allowing for prompt interventions if needed, thus maintaining the overall efficiency and effectiveness of model training.

**Figure 6:**
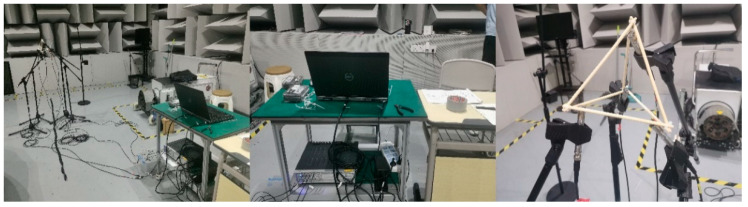
Experimental setup inside the semi-anechoic chamber.

**Figure 7:**
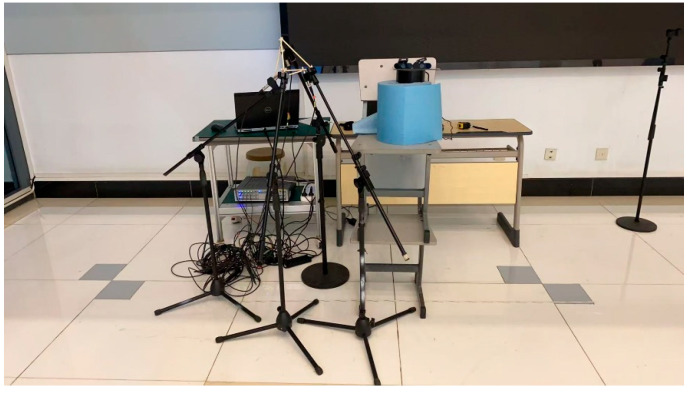
Experimental setup inside the conference room.

**Figure 8:**
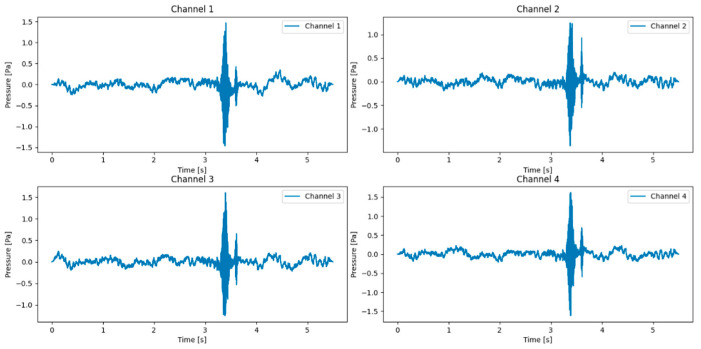
Waveform diagram of speech signal.

For validation, the proposed model was tested in acoustic environments distinct from the training environments. This ensured robustness under diverse real-world conditions. Firstly, an acoustic acquisition system was employed, combining LMS Test.Lab 2024 R2 software with high-performance acoustic microphones. The system utilized the PCB-378B02 microphone (Instrument: PCB-378B02 1/2 prepolarized free-field condenser microphone; Company name: PCB Piezotronics, Inc. (An Amphenol Company); Address: 3425 Walden Avenue, Depew, NY 14043, USA), which is highly sensitive and has a broad frequency range, capturing both weak and loud sounds with accuracy. The collected data was saved in WAV format and used as input for model testing. Then, the experimental setup in the semi-anechoic chamber and the meeting room, shown in Figure 6 and Figure 7, featured a modular stand that positioned four professional microphones in a regular tetrahedral configuration to create a microphone array. The purpose of the white frame is to more intuitively view the structure of the microphone array. To evaluate the model’s ability to localize sound sources, a loudspeaker was sequentially placed at different positions around the microphone array, with the distance from the origin ranging between 1 and 5 m., with the array center serving as the coordinate origin. The audio stimulus emitted from the speaker was recorded by the microphones, digitized using a computer, and saved in the designated audio format. The room parameters are shown in Table 5. The waveform analysis of the multi-channel audio recordings is displayed in Figure 8. While the figures show the full raw audio data, only selected portions (the audio segment between the 3rd and 4th second of the full audio) were used in the experimental analysis.

To verify the specific contributions of the residual connections and the attention mechanism in the proposed model to its performance, ablation experiments were conducted for detailed analysis. The model using only residual connections was named Without-SE, while the model using only the attention mechanism was named Without-RC. The complete model incorporating both components was designated as Proposed. Additionally, to further demonstrate the advantages of the Proposed model, comparisons were made with state-of-the-art sound source localization methods, including the SPR-PATH [26]-based approach and an end-to-end sound source localization Method in the Literature [27]. The number of parameters and inference speed for each model are summarized in Table 6.

### 3.2. Result and Analysis

#### 3.2.1. Simulated Environment Experiment

Eighty test data samples were randomly selected from the dataset to ensure that they did not participate in the training of the proposed model. The Euclidean distance between the real sound source and the predicted sound source were used as the evaluation metric for the model’s localization error. The average error of these test samples were used as the final error result, and to more intuitively display the positioning results, the localization accuracy (ACC) is defined as follows:

(4) $$ACC=\left(1-\frac{error}{D}\right)\times 100\%$$

where the error is the distance between the real sound source and predicted sound source, and D is the distance between the coordinate origin (the center of the microphone array) and the real sound source.

**Figure 9:**
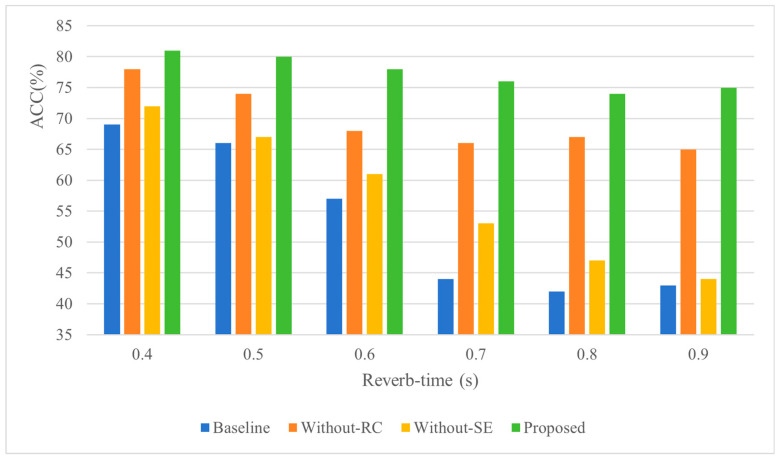
The influence of RT (60) on positioning accuracy.

Table 7 presents the localization results for all models under different reverberation times while keeping the SNR fixed at 50 dB and room size fixed at 5 × 2 × 2 m. The results show that all methods experience an increase in localization error and decrease in localization accuracy as reverberation time increases, which aligns with the consensus that the difficulty of sound source localization increases significantly with longer reverberation times. When the reverberation time was short (0.4 s), the localization errors of the four methods showed minimal differences. As the reverberation time increased to 0.9 s, significant discrepancies in error values emerged among the methods. The proposed method achieved the smallest error of 0.54 m, followed by the Without-RC method at 0.65 m, while baseline and Without-SE methods exhibited the largest errors of 0.84 m and 0.85 m, respectively. This demonstrates that the two methods incorporating SE modules exhibit significantly better anti-reverberation capabilities compared to others. This superiority stems from the mechanism’s ability to enhance the weighting of direct sound-related features while suppressing interference from reflected noise through attention mechanisms. Moreover, the self-attention mechanism in these models can focus on low-frequency components that are less affected by reverberation, thereby improving robustness in reverberant environments. The superior performance of the proposed method, achieving the minimum error among all approaches, is attributed to the synergistic combination of RC and SE modules. Although RC alone demonstrates weaker anti-reverberation capability compared to SE, their joint operation enables RC to deliver original signal information to deeper network layers while SE assists the model in distinguishing between direct sound and early reflections. This collaborative mechanism allows the model to achieve enhanced resistance to reverberation interference through complementary advantages of both components. The localization accuracy results in Figure 9 reveal that, although all methods exhibit performance degradation with increasing reverberation time, the deterioration rate of methods without SE modules is significantly faster than those incorporating SE. This observation further confirms the substantial effectiveness of SE in mitigating reverberation interference. The accelerated performance decline in SE-absent methods underscores how the attention mechanism in SE modules specifically preserves critical localization features by adaptively emphasizing direct-path components and suppressing reverberation-distorted patterns, thereby demonstrating its essential role in maintaining robustness under challenging acoustic conditions.

The results presented in Table 8 show the localization errors under varying SNR with a fixed reverberation time of 0.4 s and a room dimension of 5 × 2 × 2 m. The data indicate a consistent trend: as SNR increases, the localization errors of all methods decrease. Notably, when SNR exceeds 40 dB, the magnitude of error reduction diminishes significantly. This phenomenon occurs because noise interference in sound source localization primarily manifests as the masking of temporal and spectral features of the source signal—features that localization algorithms rely on as critical cues. As SNR increases, the masking effect of noise on these features gradually weakens. Beyond 40 dB, the signal power dominates the noise power, substantially reducing noise interference in both temporal and spectral domains, leading to stabilized localization errors despite further SNR improvements.

Regarding performance across methods, at 50 dB SNR, the baseline and Without-SE methods exhibit the largest errors (0.48 m and 0.49 m, respectively), followed by the Without-RC method (0.45 m), while the Proposed method achieves the smallest error (0.39 m). This discrepancy arises because, under low-SNR conditions, noise significantly obscures the signal’s time-frequency features, degrading the performance of feature-dependent localization algorithms. The incorporation of SE modules addresses this by dynamically reweighting spatial features—enhancing contributions from signal-dominant regions while suppressing noise-affected areas—thereby improving noise robustness. However, when noise contains frequency components similar to the source signal, SE alone becomes insufficient for effective signal-noise discrimination. Here, the RC mechanism complements SE by preserving low-level features of the original signal to prevent noise amplification in deep network layers. Simultaneously, the deeper network architecture enabled by RC extracts multi-scale features, which, combined with SE’s selective emphasis on critical patterns, further enhances the algorithm’s noise resistance. This synergistic integration of SE and RC mechanisms underpins the proposed method’s superior performance in noisy environments.

**Figure 10:**
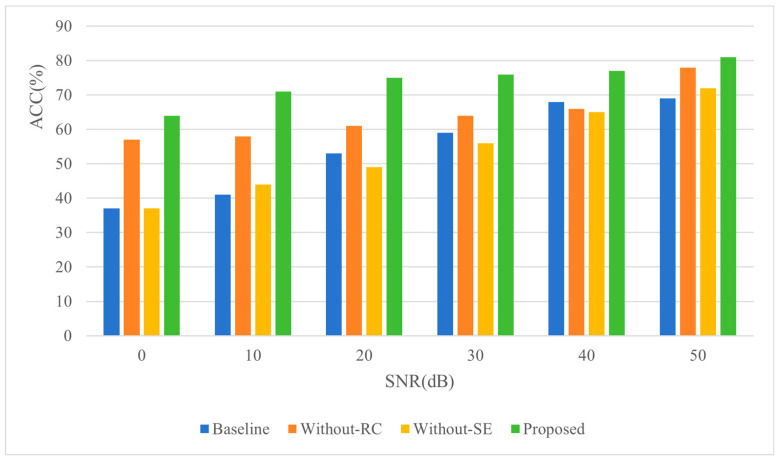
The influence of SNR change on positioning accuracy.

Figure 10 illustrates the localization accuracy under identical experimental conditions, demonstrating a trend consistent with the localization error results: accuracy progressively improves with increasing SNR. Compared to the baseline method, the proposed method achieves 11% higher accuracy at 50 dB SNR and 27% greater accuracy at 0 dB SNR, further validating its enhanced noise resistance. When benchmarked against Without-RC and Without-SE methods, the Proposed method exhibits accuracy improvements of 10% and 13% at 50 dB SNR, and 7% and 27% at 0 dB SNR, respectively. These results reinforce the significant contribution of the SE module to the algorithm’s noise robustness. Specifically, the SE mechanism amplifies discriminative acoustic features while suppressing noise-corrupted patterns through channel-wise attention, whereas the RC structure preserves critical low-level signal characteristics across network layers. Their synergistic integration enables the proposed method to maintain superior performance across diverse noise conditions, highlighting the critical role of the SE module in mitigating feature degradation caused by noise interference.

Table 9 presents the variation trends of localization errors under different room dimensions with a fixed reverberation time of 0.4 s and an SNR of 50 dB. Consistent with conventional understanding, localization errors increase with larger room dimensions. Notably, the Without-RC method exhibits significantly smaller errors compared to the baseline method as room size expands—a pattern contrasting with observations under variable SNR and reverberation time conditions. This discrepancy stems from the non-linearly increasing number of sound wave propagation paths in larger rooms, which substantially elevates the spatial complexity of acoustic models and intensifies challenges for sound source localization. The integration of RC addresses this by enabling deeper network architectures without overfitting risks, thereby enhancing the model’s fitting capacity to handle growing spatial complexity. Additionally, the SE module improves robustness against spatial scaling by focusing on direct sound features while suppressing interference from reflected sound components.

**Figure 11:**
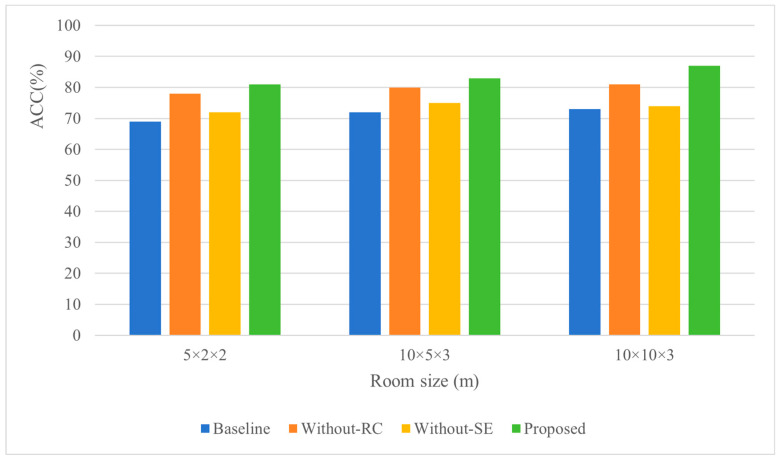
The influence of room size change on positioning accuracy.

Figure 11 illustrates the localization accuracy of all methods across varying room dimensions. Contrary to expectations, the results show an anomalous increase in localization accuracy with larger rooms rather than a decline. This phenomenon arises because, although localization errors grow with room dimensions, their rate of increase is slower than the expansion of room size. As defined by the localization accuracy formula in prior discussions, this relative relationship results in an upward trend in accuracy values. Consequently, within a certain spatial range, localization accuracy improves as distance increases. Specifically, the slower error growth relative to dimensional scaling reflects the algorithm’s ability to maintain discriminative feature extraction in larger acoustic spaces, while the SE module’s attention mechanism and RC’s hierarchical feature preservation synergistically mitigate spatial complexity effects. This underscores the importance of architectural design in balancing error propagation and spatial adaptability for robust acoustic localization systems.

#### 3.2.2. Real Physical Environment Experiment

Table 10 and Table 11 present the localization error results of the proposed method and baseline method in semi-anechoic chamber and conference room environments, respectively, with comparisons to two representative methods, SPR-PATH and Method in the Literature [27].

In the semi-anechoic chamber environment, as shown in Table 10, the proposed method achieves the smallest localization error of 0.43 m, followed by SPR-PATH (0.45 m), baseline (0.63 m), and Method in the Literature [27] (0.72 m). Regarding localization accuracy, the proposed method attains the highest accuracy of 81%, outperforming the baseline (71%), Method in the Literature [27] (67%), and SPR-PATH (61%). For inference speed, SPR-PATH demonstrates the fastest processing time at 1.2 ms, followed by Method in the Literature [27] (1.5 ms), Proposed method (3.1 ms), and baseline (6.1 ms). Overall, while SPR-PATH excels in localization error and inference speed, its poor accuracy stems from its non-end-to-end architecture, which requires preliminary feature processing to reduce model complexity. This design sacrifices accuracy for efficiency, particularly under far-field conditions. In contrast, the proposed method achieves optimal error and accuracy metrics but incurs higher computational costs due to its end-to-end design, which employs deeper networks for enhanced fitting capability.

**Figure 12:**
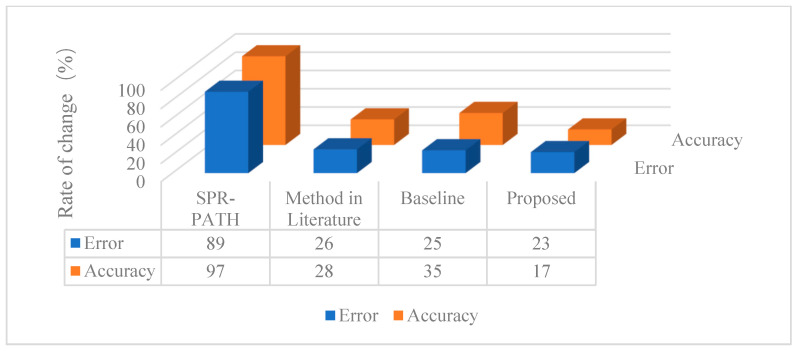
Rate of change in positioning results from semi-anechoic chamber to conference room environments.

In the conference room environment (see Table 11), all methods exhibit degraded performance compared to the semi-anechoic chamber. The proposed method maintains the smallest localization error (0.53 m), followed by baseline (0.79 m), SPR-PATH (0.85 m), and Method in the Literature [27] (0.91 m). For accuracy, the proposed method again leads with 67%, trailed by Method in the Literature [27] (48%), baseline (46%), and SPR-PATH (31%). Since acoustic environmental changes do not alter model parameters, inference speeds remain consistent across environments. Figure 12 illustrates the variation rates of localization metrics across environments, revealing stark contrasts in robustness: SPR-PATH suffers severe performance fluctuations (>80% variation rate), while the three end-to-end methods (including the proposed method) show stable variations (<30%). This highlights SPR-PATH’s sensitivity to acoustic disturbances (e.g., reverberation, noise) and underscores the inherent robustness of end-to-end approaches. Notably, the proposed method achieves the lowest variation rate among end-to-end methods, demonstrating its superior ability to mitigate reverberation and noise interference.

## 4. Conclusions

To enhance the accuracy of sound source localization, this paper addresses the challenges posed by SNR and reverberation time. Traditional signal processing algorithms often struggle in environments with high reverberation and low SNR. To overcome these limitations, the study leverages the proven effectiveness of deep learning across various domains and proposes an end-to-end sound source localization model. By integrating a frequency analysis module, an attention channel module, and a residual module, the model improves robustness against reverberation and noise. The conclusions are as follows:

(1) The ablation study results reveal that the SE module contributes more than RC to the overall algorithm performance, thereby demonstrating SE’s outstanding robustness against reverberation, noise, and other interferences.

(2) The results from both simulations and real-world experiments demonstrate that the combined use of RC and SE can further enhance the algorithm’s robustness against reverberation, noise, and other interference.

(3) The experimental findings in real-world physical environments indicate that the end-to-end sound source localization algorithm exhibits significantly superior performance compared to traditional algorithms regarding robustness against reverberation and noise. Specifically, this approach demonstrates an average improvement in performance of approximately 60%.

Despite significant advancements in sound source localization, several areas remain open for further exploration. Future research will focus on the following directions to enhance the robustness and generalizability of the proposed method:

(1) Establishment of Diverse Acoustic Environments: Future work will aim to create acoustic environments that closely resemble real-world conditions, including those similar to the training data distribution and non-stationary noise.

(2) Multi-sound Source Localization Challenge: Future research will explore the integration of video signals as complementary inputs, fusing auditory and visual modalities to enable robust multi-source localization.

(3) Moving Sound Source Localization and Tracking Problem: Future research will explore source separation approaches to achieve simultaneous localization and tracking of multiple moving sound sources.

## Figures and Tables

**Table 1:** Acoustic environment setting.

Item	Room Size (m)	SNR (dB)	Reverb-Time (s)	Noise
1	5 × 2 × 2 10 × 5 × 3 10 × 10 × 3	0	0.4	Pink noise
2		10	0.5	
3		20	0.6	
4		30	0.7	
5		40	0.8	
6		50	0.9	

**Table 2:** Microphone and environmental parameters.

Item	Value
Microphone Model	PCM378B02
Microphone Sensitivity (dBV/Pa)	−38
Microphone Frequency Response (Hz)	20–20k
Polar Pattern	Cardioid
Temperature (°C)	20~25
Humidity (%)	40~60
Atmospheric Pressure (kPa)	101~104
Wind Speed (m/s)	≤0.1

**Table 3:** The tensor shape of each layer of baseline model.

Name	Input Shape	Output Shape	Params
Input	(16,8000,4)	(16,8000,4)	-
Frequency analysis	(16,8000,4)	(16,3872,64)	65,600
Cross-channel feature extraction	(16,3872,64,1)	(16,3872,16,64)	2368
Deep feature extraction	(16,3872,16,64)	(16,3870,4,64)	12,352
Combined feature extraction	(16,3870,4,64)	(16,64)	63,410,240
Output	(16,64)	(16,3)	195

**Table 4:** The tensor shape of each layer of proposed model.

Name	Input Shape	Output Shape	Params
Input	(16,8000,4)	(16,8000,4)	-
Frequency analysis	(16,8000,4)	(16,8000,64)	960
Cross-channel feature extraction	(16,8000,64)	(16,8000,128)	24,960
Max-Pooling 1-D	(16,8000,128)	(16,2666,128)	-
Rese Block × 5	(16,2666,128)	(16,10,128)	625,536 × 5
Global-Max-Pooling	(16,10,128)	(16,128)	-
Combined feature extraction	(16,128)	(16,256)	33,536
Output	(16,256)	(16,3)	771

**Table 5:** Room parameters.

Room Type	Size (m)	SNR (dB)	Reverb-Time (s)
Semi-anechoic chamber	6 × 7 × 4	16	0.1
Conference room	8 × 9 × 4	40	0.8

**Table 6:** Params and inference speed of models.

Model	Params (Million)	Inference Speed (ms)
Baseline	6.3	6.13
SPR-PATH	0.8	1.21
Method in the Literature [27]	1.2	1.54
Without-SE	2.6	2.66
Without-RC	1.8	2.41
Proposed	3.1	3.11

**Table 7:** The influence of RT (60) on positioning error (m).

Reverb-Time (s)	0.4	0.5	0.6	0.7	0.8	0.9
Baseline	0.48	0.47	0.62	0.69	0.81	0.84
Without-RC	0.45	0.49	0.51	0.64	0.63	0.65
Without-SE	0.49	0.50	0.63	0.72	0.80	0.85
Proposed	0.39	0.45	0.39	0.48	0.52	0.54

**Table 8:** The influence of SNR change on positioning error (m).

SNR (dB)	0	10	20	30	40	50
Baseline	0.73	0.69	0.69	0.58	0.49	0.48
Without-RC	0.68	0.64	0.61	0.52	0.44	0.45
Without-SE	0.75	0.71	0.68	0.56	0.51	0.49
Proposed	0.52	0.48	0.45	0.43	0.38	0.39

**Table 9:** The influence of room size change on positioning error (m).

Room Size (m)	5 × 2 × 2	10 × 5 × 3	10 × 10 × 3
Baseline	0.48	0.69	0.88
Without-RC	0.45	0.57	0.77
Without-SE	0.49	0.59	0.75
Proposed	0.39	0.51	0.63

**Table 10:** Localization result in semi-anechoic chamber.

Method	Error (m)	Accuracy (%)	Inference Speed (ms)
SPR-PATH	0.45	61	1.22
Method in the Literature [27]	0.72	67	1.53
Baseline	0.63	71	6.11
Proposed	0.43	81	3.12

**Table 11:** Localization result in meeting room.

Method	Error (m)	Accuracy (%)	Inference Speed (ms)
SPR-PATH	0.85	31	1.23
Method in the Literature [27]	0.91	48	1.55
Baseline	0.79	46	6.17
Proposed	0.53	67	3.11

## Data Availability

The data that support the findings of this study are available on request from the corresponding author.
